# Chronic stress and intestinal barrier dysfunction: Glucocorticoid receptor and transcription repressor HES1 regulate tight junction protein Claudin-1 promoter

**DOI:** 10.1038/s41598-017-04755-w

**Published:** 2017-07-03

**Authors:** Gen Zheng, Gordon Victor Fon, Walter Meixner, Amy Creekmore, Ye Zong, Michael K. Dame, Justin Colacino, Priya H. Dedhia, Shuangsong Hong, John W. Wiley

**Affiliations:** 1 University of Michigan Medical School, Division of Gastroenterology, Department of Internal Medicine, Ann Arbor, 48109 USA; 2 University of Michigan Medical School, Department of Computational Medicine and Bioinformatics, Ann Arbor, 48109 USA; 3 Beijing Friendship Hospital Affiliated to Capital Medical University, Department of Gastroenterology and Hepatology, Beijing, 100050 China; 4 University of Michigan Medical School, Department of Pathology, Ann Arbor, 48109 USA; 5 University of Michigan School of Public Health, Department of Environmental Health Sciences, Ann Arbor, 48109 USA; 6 Department of Surgery, University of Michigan, Ann Arbor, MI 48109 USA

## Abstract

Chronic stress and elevated glucocorticoid hormone are associated with decreases in the intestinal epithelial tight junction protein claudin-1 (CLDN1). Human/rat *CLDN1* promoters contain glucocorticoid response elements (GREs) and adjacent transcription repressor HES1 binding N-boxes. Notch signaling target HES1 expression was high and glucocorticoid receptor (NR3C1) low at the crypt base and the pattern reversed at the crypt apex. Chronic stress reduced overall rat colon HES1 and NR3C1 that was associated with CLDN1 downregulation. Chromatin-immunoprecipitation experiments showed that HES1 and NR3C1 bind to the *CLDN1* promoter in rat colon crypts. The binding of NR3C1 but not HES1 to *CLDN1* promoter significantly decreased in chronically stressed animals, which was prevented by the NR3C1 antagonist RU486. We employed the 21-day Caco-2/BBe cell model to replicate cell differentiation along the crypt axis. HES1 siRNA treatment early in differentiation increased CLDN1. In contrast, stress levels of cortisol decreased CLDN1 in late differentiation stage but not in the early stage. HES1 was high, whereas NR3C1 and CLDN1 were low in the early stage which reversed in the late stage, e.g. HES1/NR3C1 binding to *CLDN1* promoter demonstrates a dynamic and reciprocal pattern. These results suggest that chronic stress impairs colon epithelium homeostasis and barrier function via different mechanisms along the crypt axis.

## Introduction

Intestinal epithelial tight junction proteins contribute to intestinal barrier function via their role in regulating paracellular permeability^1^. Impaired intestinal barrier function involving increased epithelial paracellular permeability has been reported in several gastrointestinal disorders including Irritable Bowel Syndrome (IBS)^2^. We reported previously that elevation in serum corticosterone mediates chronic stress induced decrease in specific intestinal epithelial tight junction proteins that was associated with an increase in epithelial paracellular permeability in the colon but not the jejunum in a validated chronic, intermittent water avoidance (WA) stress rat model^3^. A maternal-pup deprivation rat model of early life stress demonstrated similar responses^4^. Regional profiling of epithelial tight junction proteins in the intestine revealed that the colon has the highest levels of the tight junction protein claudin 1 (CLDN1) which is responsible for “tightening” the tight junctions^5^. It is noteworthy that baseline expression of the glucocorticoid signaling target NR3C1 (GR) is significantly higher in colon epithelial cells compared to the jejunum^3^. Chronic stress and treatment of control rats with a chronic stress level of corticosterone led to the reduction in colon lumen epithelial cell CLDN1 compared to controls^3^. It is unknown if NR3C1 directly regulates colon *CLDN1* transcription through binding to glucocorticoid response elements (GREs) on the *CLDN1* promoter at its transcription start site (TSS).

The intestinal epithelium consists of millions of contiguous crypts containing stem cells at their base which generate cells responsible for a variety of functions including secretion and absorption^6^. Basic helix loop helix (bHLH) transcription repressor HES1 (hairy and enhancer of split-1) is a ubiquitous cell marker of neuronal differentiation^7^. HES1 is also required for differentiation of colon epithelium enterocytes^8–10^. HES1 positive cells are present at the base of colon crypts, but this expression pattern is altered under specific disease conditions^8^. In contrast, apical and luminal colon epithelial cells express significantly higher NR3C1 mRNA compared to basal regions of crypts^11^. A “HES1-NR3C1 axis” was identified in elevated glucocorticoid-induced fatty liver disease, e.g. NR3C1 directly inhibited *HES1* promoter transcription via negative GRE^12^. Luciferase reporter plasmids with HES1 binding N-boxes and NR3C1 binding GREs similar to rat *CLDN1* promoter demonstrate cooperative transcription regulation by HES1 and NR3C1, without HES1-NR3C1 protein-protein interaction^13^. However, over-expression of CLDN1 in transgenic mouse colon resulted in increased HES1 mRNA levels and impaired colon epithelial homeostasis^14^. These observations suggest that a dynamic equilibrium between HES1 and CLDN1 with feedback pathways may be involved in colon epithelial homeostasis. For example, reciprocal distribution of HES1 & NR3C1 along the colon crypts may regulate CLDN1 and colon barrier function in a specific manner that is dependent on stages of differentiation. Notch-1 signaling was reported to regulate the colon barrier function, whether if its signaling target HES1 is involved is still not clear^14, 15^.

We examined the hypothesis that CLDN1 expression in colon epithelial cells is regulated by NR3C1 and HES1 via their corresponding GRE and N-box elements on the *CLDN1* gene promoter, and that chronic stress alters CLDN1 transcription via these transcription factor binding DNA elements on the *CLDN1* promoter. We believe that this is the first study to examine a potential role for a HES1-NR3C1 regulatory axis on CLDN1 expression in colon epithelial cells in healthy controls, and a validated rodent model of chronic stress, as well as provide supportive data that a similar pattern of expression occurs in human colon crypts.

## Results

### Differential distribution of NR3C1 and HES1 in human and rat colon crypts

**Figure 1:**
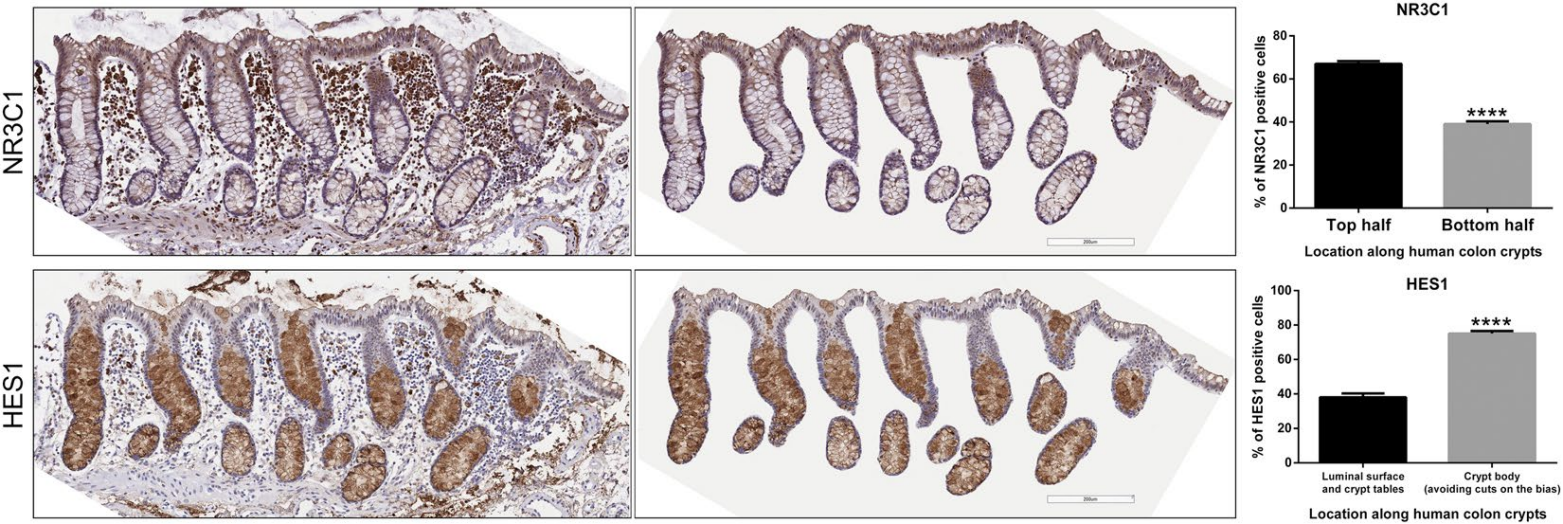
Immunohistochemistry analysis of human colon crypts. Healthy control human colon samples are fixed for Immunohistochemistry analysis. HES1 positive cells showed basal localization and NR3C1 positive cells showed apical localization along crypt axis. (****P < 0.0001; n = 24 or 14 crypts).

We performed immunohistochemistry (IHC) analysis of healthy human colon tissue with IHC validated NR3C1 and HES1 antibodies. NR3C1 immunostaining signals were positive at the apical side of human colon crypts, and HES1 immunostaining signals were positive at the basal side of the human colon crypts (Fig. 1). Tissue was fixed immediately in formalin soon after death which was important to detect and demonstrate reproducibility in staining patterns. All four donors displayed this pattern but the gradient of expression was more pronounced in two of the donors. Only intact (full-length) mucosal crypts of 225 μm or greater length were quantified. Specifically, colon tissue samples were collected from: 1. 33-year-old female; 2. 56-year-old female; 3. 29-year old female and 4. 55-year-old male. The number of crypts analyzed from each donor ranged from 14 to 24 crypts. Overall, HES1-positive cells dominated the basal proliferating cells and NR3C1 dominated the apical differentiated cells. The top half of the crypts demonstrated 67.0 ± 1.3% NR3C1 positive cells which was significantly higher than the bottom half of the crypts (39.0 ± 1.3%, P < 0.0001). At the luminal surface, 38.0 ± 1.3% of cells were HES1-positive which was significantly lower than HES1-positive cells in the crypt body (75.0 ± 2.0%, P < 0.0001). NR3C1 tended to dominate the apical differentiating/differentiated cells. A similar HES1/NR3C1 distribution pattern was observed in rat colon crypts (Figure S1). We noticed that rat and human *CLDN1* conserved promoter regions contain putative NR3C1 binding GRE element and HES1 binding N-boxes elements suggesting dual regulation of *CLDN1* promoter by NR3C1 and HES1 (Figure S2).

### Identification of GREs on rat *CLDN1* promoter

**Figure 2:**
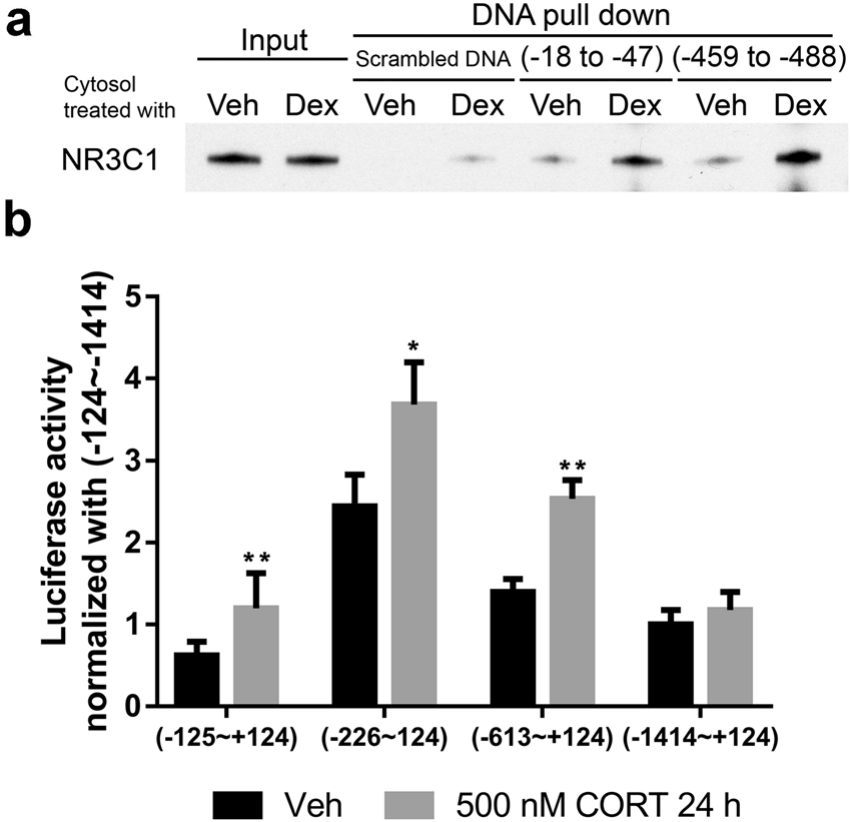
Identification of rat *CLDN1* promoter GREs. (**a**) DNA pulldown for NR3C1 with control, GRE1 (−18 to −47) and GRE2 (−459 to −488) DNA from the vehicle and dexamethasone-activated cytosol. Both of the predicted GREs showed higher NR3C1 binding after dexamethasone (Dex) treatment compared to the vehicle control. (**b**) Rat *CLDN1* promoter activity in FRC/TEX cells in response to glucocorticoid treatment. Rat *CLDN1* promoter truncation plasmid −226 containing the first GRE (−18 to −47) had highest transcription activity. Corticosterone (500 nM) treatment enhanced CLDN1 transcription by 50.9 ± 21.2% in this truncation (*P* < 0.05). (n = 4; two-tailed test; *P < 0.05; **P < 0.01).

We used early stage undifferentiated Caco-2/BBe cytosol to test specific NR3C1 binding to GREs via *in vitro* DNA pull-down assays. Dexamethasone (DEX) was used to activate cytosol NR3C1 receptor and its DNA binding activity. As shown in the Fig. 2a, predicted GREs (−18 to −47 and −459 to −488) showed stronger NR3C1 binding capability compared to the scrambled control. The amounts of NR3C1 binding to these two predicted GREs were significantly higher after dexamethasone treatment, suggesting that these two GREs on the *CLDN1* promoter can be actively regulated by glucocorticoids. In order to test GRE transactivation activity of the predicted GREs on rat *CLDN1* promoter, we employed the rat colon epithelial cell line FRC/TEX CL D [D/WT] that expresses a high-level of NR3C1. Four truncated plasmids containing one or two predicted GRE sequences were transfected. The truncated plasmid (−226 to +124) containing the first upstream GRE (−18 to −47) demonstrated the highest transcription activity. Exposure to the NR3C1 agonist corticosterone enhanced *CLDN1* transcription activity in all the four truncated plasmids, indicating that predicted rat *CLDN1* promoter GRE elements respond to glucocorticoid (Fig. 2b).

### Chronic stress induces down-regulation of CLDN1 and NR3C1 in rat colon luminal epithelial cells

**Figure 3:**
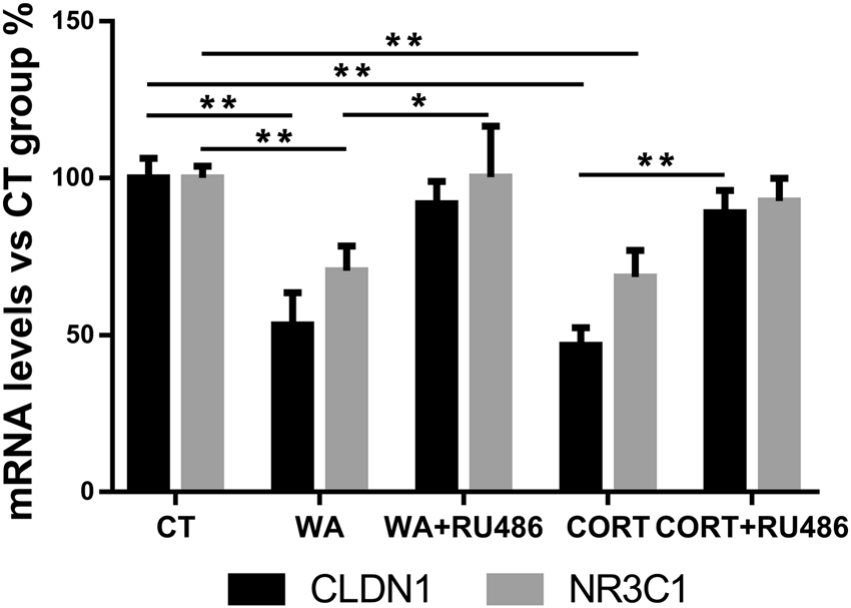
Realtime PCR analysis of mRNA levels in rat colon luminal epithelium from the following groups: control (CT), water avoidance (WA) stress, WA stress with NR3C1 antagonist RU486 intervention (WA + RU486), corticosterone (CORT) treatment, and CORT treatment plus RU486 intervention (CORT + RU486). Chronic WA stress and CORT treatments significantly down-regulated CLDN1 mRNA levels and concurrent RU486 treatment during the stress phase prevented down-regulation of CLDN1 mRNA levels. Chronic WA stress and CORT treatments significantly down-regulated NR3C1 mRNA levels and concurrent RU486 treatment demonstrated modest preventions. (n = 3, 4; two-tailed test; *P < 0.05; **P < 0.01).

We reported previously that chronic WA stress and corticosterone treatment decreased colon lumen CLDN1 protein expression in the rat, which was prevented by the NR3C1 antagonist RU486^3^. It is unknown whether chronic stress affects tight junction protein expression in colon epithelial cells at the transcription (mRNA) level. We performed real-time PCR analysis to examine the changes of mRNA levels of CLDN1 and NR3C1 in stressed rat colon luminal epithelial cells harvested by scraping. CLDN1 mRNA was down-regulated [46.6 ± 10.1% (n = 4, *P* < 0.01) and 53.2 ± 5.7% (n = 3, *P* < 0.01)] by WA stress and corticosterone injections respectively. RU486 interventions prevented these decreases (Fig. 3). NR3C1 mRNA was down-regulated [29.6 ± 7.9% (n = 4, *P* < 0.01)] in the WA-stressed rats and [31.6 ± 8.6% (n = 3, *P* < 0.01)] in the corticosterone-treated healthy rats. These results were consistent with the changes in protein levels reported previously^3^.

### Chronic stress decreased NR3C1 but not HES1 binding to *CLDN1* promoter in rat colon crypts

**Figure 4:**
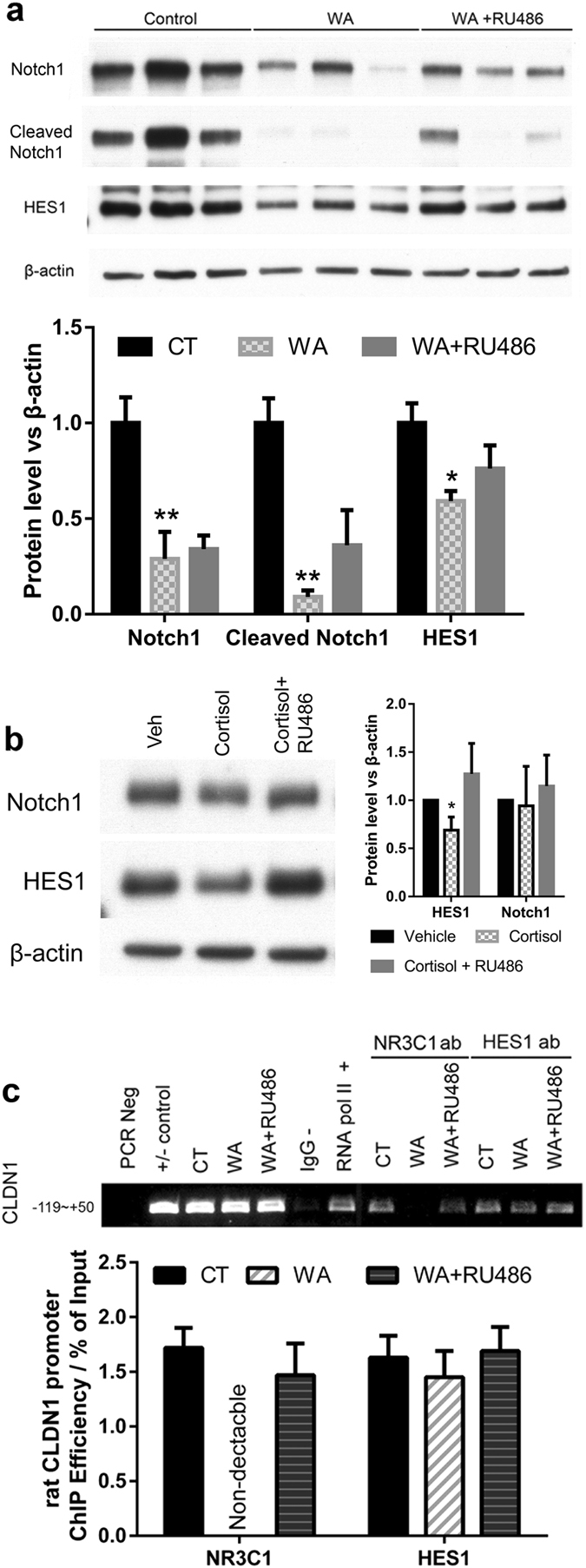
CLDN1, NR3C1, and HES1 in WA stress rat crypts. (**a**) Western blotting analysis of colon crypts isolated from control, WA and WA + RU486 rats. Notch signaling target HES1 was down-regulated in WA stress rats (*P* < 0.05) and RU486 intervention modestly affected HES1 protein levels (P = 0.19). (n = 3; *P < 0.05). Significant down-regulation of Notch1 and cleaved Notch1 protein levels in the WA stressed rat colon crypts are also observed (n = 3; **P < 0.01). (Pre-cropped blots are included in a Supplementary Information.) (**b**) Caco2/BBe cells are treated with 500 nM cortisol or 500 nM cortisol + 500 nM RU486 24 h later seeded with 50–60% density, cells are harvested after 24 h treatment for Western blotting analysis. HES1 was showed 31.1 ± 7.9% down-regulation upon cortisol treatment (n = 3; *P < 0.05). (**c**) Chromatin Immunoprecipitation (ChIP) analysis of the interaction between rat *CLDN1* gene promoter and NR3C1 or HES1 in rat colon crypts isolated from control (CT), WA stress rats, and stressed rats with RU486 intervention (WA + RU486). PCR primers targeting (−119 ~ +50) of rat *CLDN1* promoter was used to detect immunoprecipitated product. ChIP analysis showed that both NR3C1 and HES1 bind to *CLDN1* promoter region containing GREs and N-boxes. A significant decrease of NR3C1 binding to *CLDN1* promoter was observed in colon crypts from WA stressed rats, and RU486 intervention prevented this change. (n = 3; *P* < 0.05).

Increased colon permeability in WA stressed rats was observed in our previous study, this effect could be mimicked by corticosterone and prevented by glucocorticoid antagonist RU486^3^. Notch1 was reported to regulate the colon barrier function, whether if Notch signaling target HES1 relatable by glucocorticoid is involved in stress impaired colon barrier function is still unknown^12, 15^. In enriched colon crypt preparations from WA stressed rats prepared with the same condition, HES1 protein levels were down-regulated (40.8 ± 5.3%) compared with controls (*P* < 0.05; n = 3) as shown in Fig. 4a. Down-regulation of Notch1 [70.9 ± 14.0% (n = 3, *P* < 0.01)] & Cleaved Notch1 [﻿91.3 ± 3.4%﻿ (n = 3, *P* < 0.01)] protein levels are also observed which was correlated with impaired colon barrier function^15^. Significant down-regulation of HES1 in undifferentiated Caco2/BBe cells upon 500 nM cortisol 24 h treatment is observed [31.1 ± 7.9% (n = 3; *P < 0.05)], this effect is consistent with what was observed in other cell models^12, 13^ (Fig. 4b). To test whether NR3C1 directly regulates CLDN1 expression, we performed ChIP analysis using colon crypts isolated from WA stressed and control animals. As shown in Fig. 4c, NR3C1 binding to the *CLDN1* promoter decreased to undetectable levels in colon crypts in stressed rats (n = 3, *P* < 0.01). Treatment with the NR3C1 antagonist RU486 during the stress phase significantly prevented this reduction (n = 3, *P* < 0.01). In contrast, HES1 binding to *CLDN1* promoter did not show significant changes (Fig. 4c). These data indicate that NR3C1 and HES1 bind to the *CLDN1* promoter in rat colon crypts as predicted.

### HES1 inhibits and cortisol promotes CLDN1 expression in undifferentiated Caco-2/BBe cells

**Figure 5:**
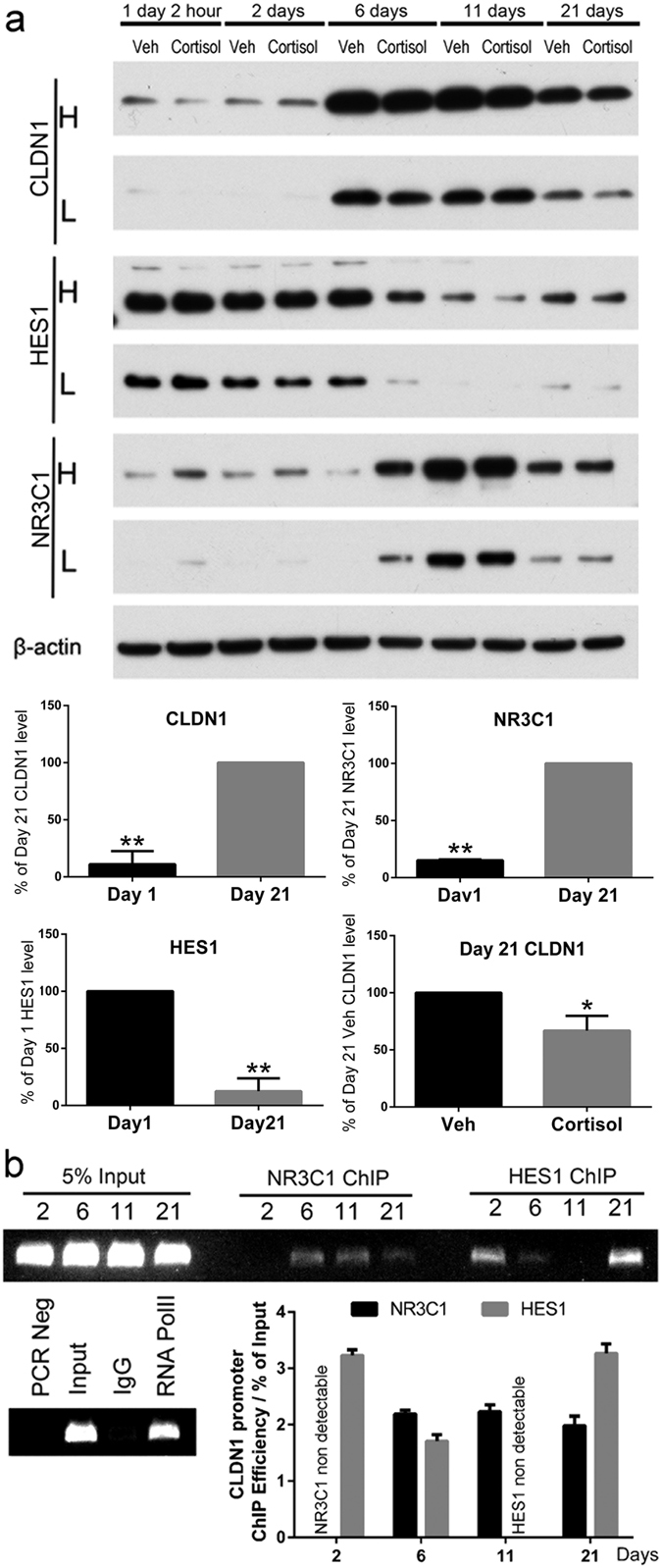
Dynamic expression of CLDN1, HES1 and NR3C1 and protein-DNA interaction in Caco-2/BBe cells during 21-day differentiation period. (**a**) Fresh medium supplied with vehicle or cortisol (500 nM) were added to the plates 24 h after Caco-2/BBe cells were seeded and then replaced every 3 days. Cells were harvested after 2 h cortisol treatment or 2, 6, 11, 21 days after seeding. High (H) exposure and low (L) exposure of X-ray films during Western blot were conducted in order to show the complete dynamic range of protein expression levels. (n = 3; two-tailed test; *P < 0.05; **P < 0.01) (Pre-cropped blots are included in a Supplementary Information). (**b**) ChIP analysis of human *CLDN1* promoter region using HES1 and NR3C1 antibodies. Binding of transcription suppressor HES1 and transcription activator NR3C1 to *CLDN1* promoter showed pattern correlated with CLDN1 protein levels. NR3C1 binding to the *CLDN1* promoter is non-detectable at day 1 whereas NR3C1 protein level is the lowest. HES1 binding to the *CLDN1* promoter is non-detectable at day 11 whereas HES1 level is the lowest (*P* < 0.01, n = 3).

**Figure 6:**
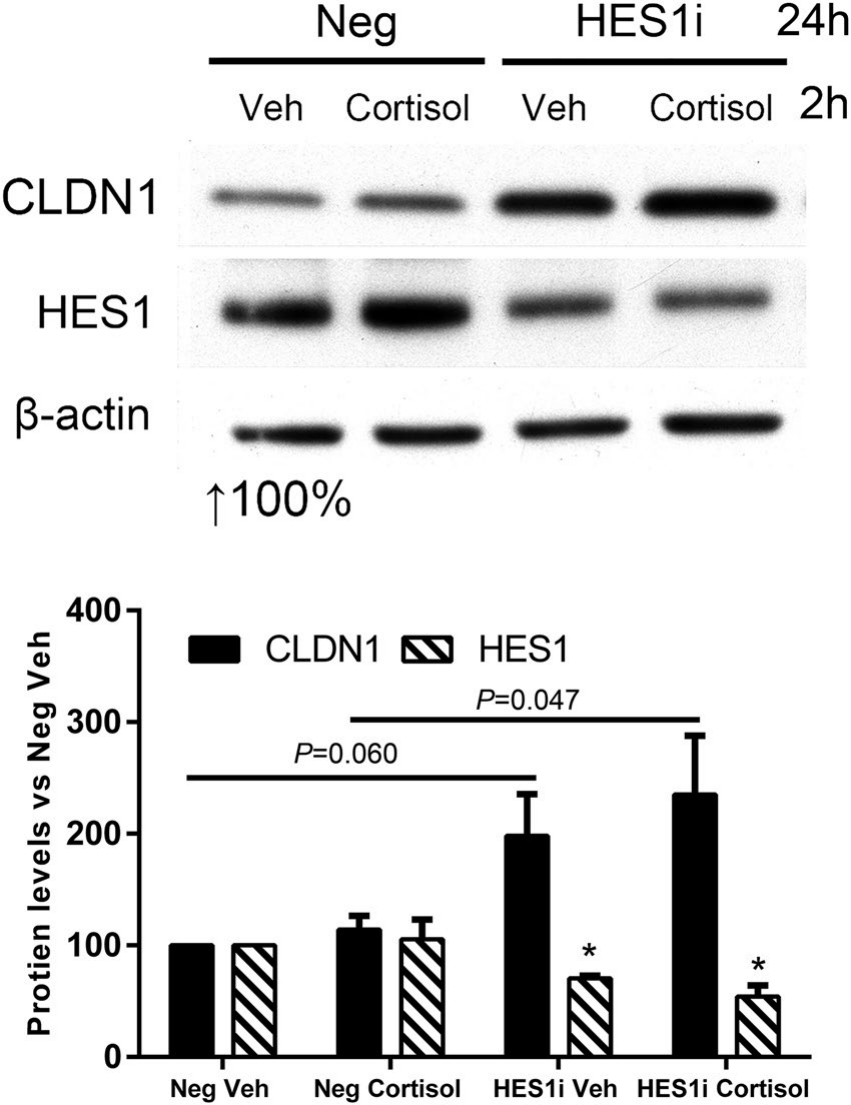
Effects of HES1 silencing on CLDN1 expression in non-differentiated Caco-2/BBe cells. Caco-2/BBe cells were seeded in 6 well plates with 50–60% density and transfected with HES1 siRNA or negative control siRNA. 24 h after transfection, cells were treated with vehicle (Veh) or 500 nM cortisol for 2 h. Both Silencing of transcription repressor HES1 and treatment of cortisol showed the effectiveness of upregulating CLDN1 levels, while cortisol treatment for 2 h significantly increased CLDN1 expression (*P* < 0.05). (n = 3; two-tailed test; *P < 0.05) (Pre-cropped blots are included in a Supplementary Information).

Undifferentiated Caco-2/BBe cells express a high level of HES1 and low levels of CLDN1 & NR3C1 as shown in Fig. 5a. We performed HES1 silencing in undifferentiated Caco-2/BBe cells to examine the regulatory role of HES1 on CLDN1 expression. After treatment with HES1 siRNA for 24 h, HES1 expression was significantly decreased 29.4 ± 2.3% in the vehicle group (*P* < 0.01; n = 3), whereas CLDN1 protein expression increased (98.2 ± 37.4%; P = 0.058; n = 3). The increase in CLDN1 level (135.3 ± 52.4%, *P* = 0.047) by HES1 silencing was significant in 2 h cortisol-treated samples. This result suggests that HES1 suppresses *CLDN1* promoter GRE in undifferentiated Caco-2/BBe cells (Fig. 6). Acute cortisol treatment alone did not affect CLDN1 expression significantly in either HES1 siRNA-treated cells or vehicle-treated cells. These observations support the interpretation that the high level of HES1 in undifferentiated Caco-2/BBe cells inhibits CLDN1 expression which can be activated by exposure to glucocorticoid when HES1 inhibition is removed. This phenomenon conforms to a genome-wide HES1-NR3C1 dual regulation mechanism^13^.

### Caco-2/BBe cell differentiation is associated with reciprocal changes in HES1 and CLDN1 expression

The 21-day differentiated Caco-2 cell enterocyte monolayer is the most widely used *in vitro* cell model to study human colon epithelial cell barrier function^16^. Caco-2/BBe cells divided and reached the confluency between day 3 and 5 under the culture conditions employed in this study. Post-confluence differentiation of Caco-2 cells demonstrated similarity to intestinal epithelial cell terminal differentiation along crypt axis^17^. The protein expression levels of CLDN1, HES1 and NR3C1 displayed a broad dynamic range during the 21-day differentiation period. HES1 levels were highest while CLDN1 and NR3C1 levels were lowest during the first two days of cell culture (Fig. 5a). After reaching confluency (day 6 in culture), robust CLDN1 up-regulation and HES1 down-regulation were observed. NR3C1 expression increased significantly in cells after 11 days in culture. On day 1, the levels of CLDN1 and NR3C1 were 11.0 ± 6.7% and 15.2 ± 0.4% (*P* < 0.01; n = 3), respectively compared to the levels measured at day 21. After 21 days differentiation, HES1 level decreased to 12.4 ± 6.6% (*P* < 0.01; n = 3) compared to day 1. Treatment of Caco-2/BBe cells with a chronic stress level of cortisol during the 21-day culture period significantly decreased CLDN1 expression [33.2 ± 7.5% (*P* < 0.05; n = 3)] as shown in Fig. 5a, similar to what we observed in the colon epithelium from the rats^3^.

We next conducted ChIP studies to examine whether HES1 and NR3C1 directly regulate CLDN1 expression through interacting with the *CLDN1* promoter in Caco-2/BBe cells. As shown in Fig. 5b, HES1 binding to *CLDN1* promoter was high on day 2 in Caco-2/BBe cells and decreased at day 6 and day 11. In contrast, NR3C1 binding to *CLDN1* promoter was undetectable at day 2 and significantly increased at day 6, 11 and 21. The level of HES1 binding to the *CLDN1* promoter was high at day 21 compared to day 11, although the HES1 expression level was lower compared to day 2 & 6, but higher than day 11.

We hypothesized that the inconsistency of the Caco-2 monolayer model may be related to the culture conditions ^15^. To test this hypothesis, we compared Caco-2/BBe cells grown to 50–60% confluence versus 80% confluence for 10 passages in the absence and presence of a chronic stress level (500 nM) cortisol. Increased transepithelial electrical resistance (TEER) values were observed in the lower density trained cells, and cortisol treatment produced a similar effect. Both low density and cortisol treated cells expressed less HES1 which correlated with impaired colon barrier function (Figure S3)^8^. We also examined the effect of changing the medium with vehicle or 500 nM cortisol every 2 days instead of 3 days. Western immunoblot analysis revealed altered expression of CLDN1, HES1 & NR3C1 with 2-day compared to 3-day intervals during the 21-day differentiation process (Figure S4). These data suggest that both HES1 and cortisol signaling regulate a dynamic equilibrium between proliferation and differentiation in colon epithelium enterocytes.

### Quantification of nuclear HES1 protein at day 1 and 21 Caco-2/BBe cell differentiation

**Figure 7:**
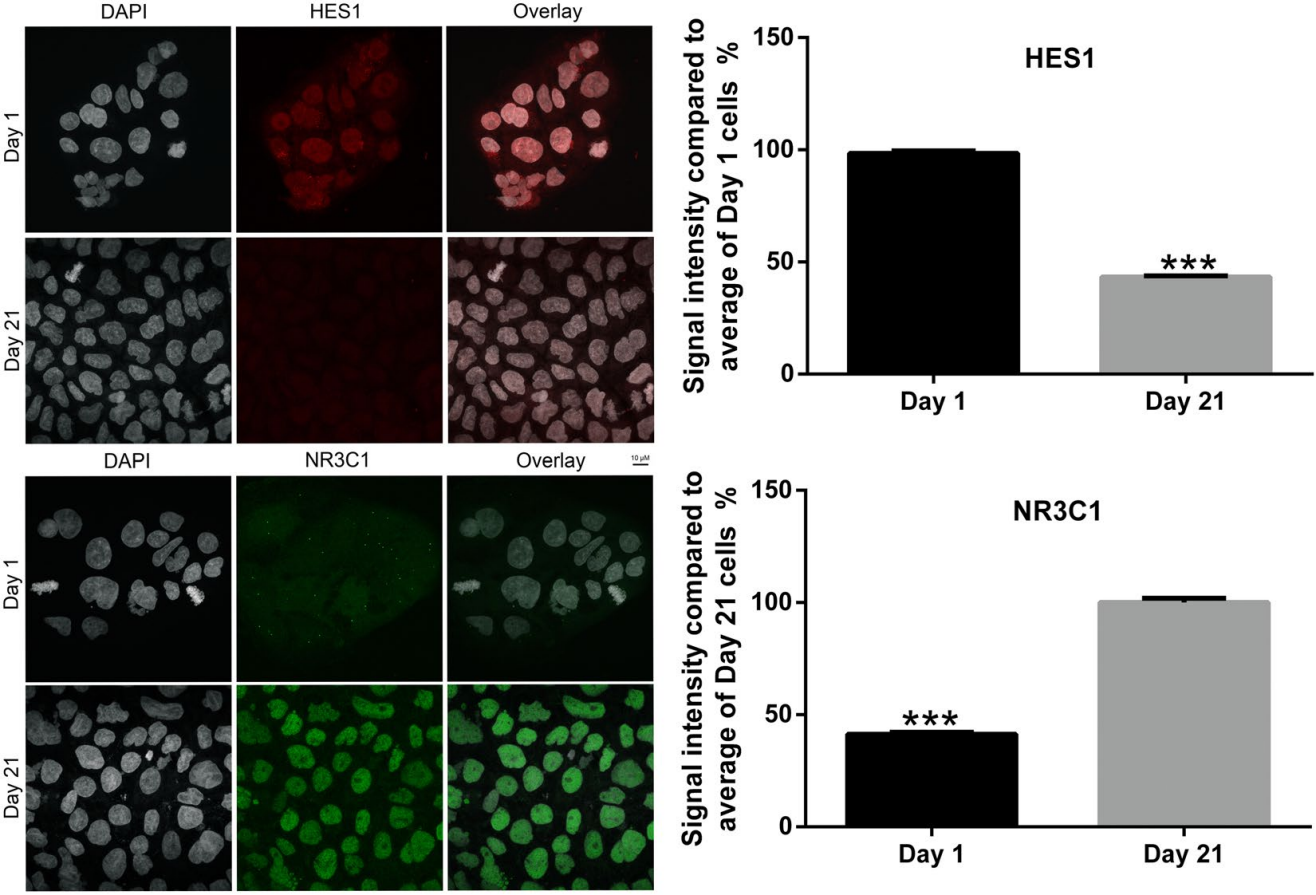
Single-cell resolution quantification of HES1 and NR3C1 proteins in Caco-2/BBe cells before and after 21-day differentiation. Caco-2/BBe cells were fixed at day 1 and day 21 of 21-day differentiation and immuno-stained with HES1 and NR3C1 antibodies for confocal imaging. 100 cells from each group were randomly selected for nucleus immunofluorescence signal quantification. Day 21 cells showed significantly lower nucleus HES1 signal (44.0 ± 0.1%) compared to day 1 cells. Day 1 cells showed only 41.3 ± 0.1% nucleus NR3C1 signal compared to day 21 cells. (n = 100; ***P < 0.001).

We used HES1 and NR3C1 antibodies to label HES1 and NR3C1 proteins for confocal imaging and performed quantification at single-cell resolution. HES1 and NR3C1 protein signals within the nucleus of day 1 & day 21 cells were quantified (Figure S5 and Fig. 7). The signal intensity of HES1 in day 21 cells was 44.0 ± 0.1% compared to day 1 cells (P < 0.001; n = 100). Day 1 cells demonstrated 41.3 ± 0.1% nuclear NR3C1 signal intensity compared to day 21 cells (P < 0.001; n = 100) (Fig. 7). This data complements what was observed with western blotting assay (Fig. 5a).

## Discussion

We studied the novel dual regulation of the *CLDN1* promoter by the transcription factors HES1 and NR3C1 that demonstrate reciprocal distribution along human/rat colon crypt axis. We confirmed the predicted NR3C1 and HES1’s binding sites on rat *CLDN1* promoter, which is homologous to the human *CLDN1* promoter. *In vitro* experiments confirmed that treatment with glucocorticoid-activated NR3C1, promoted *CLDN1* promoter transcription, and HES1 inhibited CLDN1 expression in undifferentiated Caco-2/BBe colon epithelial cells. Chronic WA stress down-regulated Notch1 & HES1 levels which were high in colon crypt proliferating enterocytes and down-regulated CLDN1 & NR3C1 levels which were high in terminal differentiated HES1 negative colon luminal epithelial cells. Time course *in vitro* studies with Caco-2/BBe cells followed for 21 days during differentiation revealed a progressive decrease in HES1 protein expression and a reciprocal increase in CLDN1 protein levels. *HES1* and *CLDN1* demonstrated opposite and coordinated gene expression response to stress levels of cortisol. Differential, reciprocal distribution of NR3C1 (low expression at the crypt base and high levels at crypt apex and luminal epithelial cells) and HES1 (high expression at crypt base and low levels at crypt apex and luminal epithelial cells) in rat and human colon crypts suggest dynamic dual regulation of human *CLDN1* promoter by NR3C1 and HES1.

We compared different techniques to harvest and enrich colon epithelial cells. The manual scrapping of the colon lumen to isolate epithelial cells demonstrated inconsistency in obtaining preparations containing a sufficient number of crypt bases for some experiments. We also employed treatment with 4 mM EDTA to isolate colon crypts which generated a greater number of HES1-positive proliferating cells present at the crypt base. This technique is used to generate intact crypts for *in vitro* studies^18, 19^. However, the method disrupts apical tight junction protein complexes, e.g. exposure to 2 µM EDTA decreased differentiated Caco-2 monolayer epithelium trans-epithelial electrical resistance (TEER) in 10 min and disrupted the CLDN1 network of intercellular junctions^20^. Therefore, we employed each technique where appropriate for our experimental objectives. We also observed variability in the differentiation of Caco-2/BBe cells during the 21-day observation period that was improved by sub-culturing the cells at 50–60% confluence instead of the conventional 80% confluence. We hypothesize that the improved consistency in cell differentiation is related to reducing contact inhibition triggered by confluence. Specifically, low-density trained cells demonstrated improved synchronicity and formed more homogeneous and polarized cell monolayers with higher TEER. These cells also demonstrated a more consistent enterocyte-like gene expression profile^16^.

**Figure 8:**
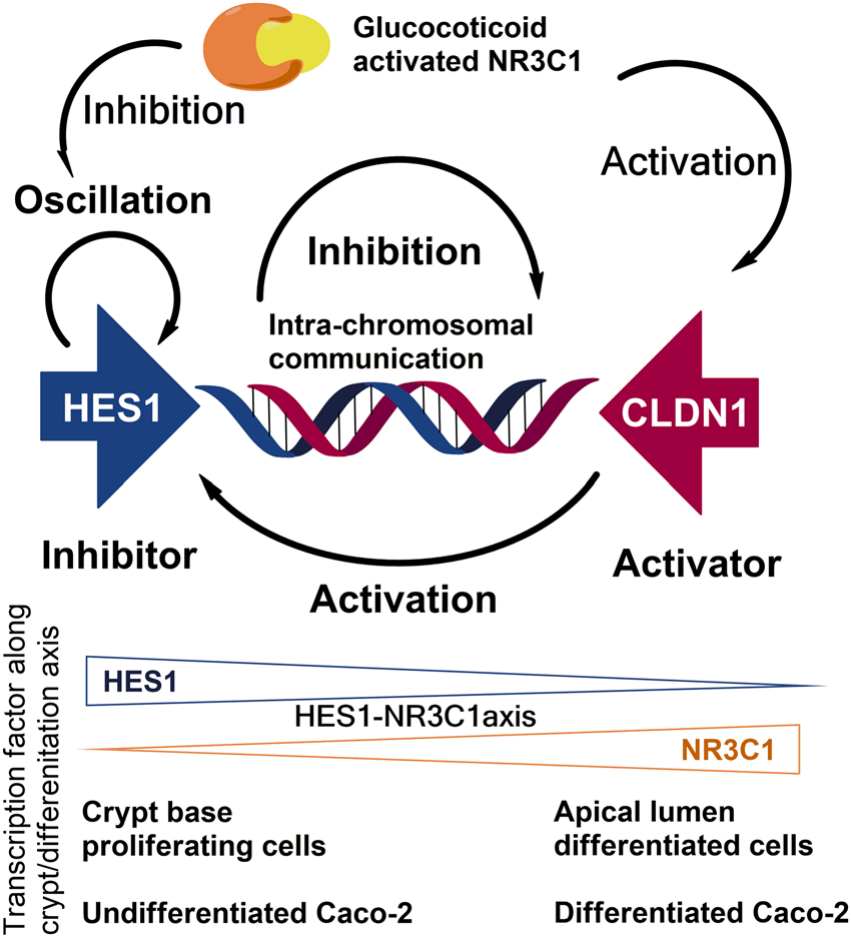
Proposed model for regulatory circuits of HES1-CLDN1 along colon crypt axis. Regulatory circuits of HES1-CLDN1 pathways are similar to that of colon & neuroectoderm patterning Notch–Delta inhibitor-activator pair. Potential chromosomal intra-communication between *HES1* and *CLDN1* coded close and oppositely on human chromosome 3 made them candidates to test Alan Turing’s 1952 gene morphogen hypothesis. Glucocorticoid activated NR3C1 inhibits *HES1* promoter and activate *CLDN1* promoter via GRE elements. Chronically elevated glucocorticoid levels may impair colon epithelium barrier function via HES1-NR3C1 axis. This model is consistent with an activator/inhibitor gene morphogen pair, originally proposed by Alan Turing^23, 26^ (refer to Discussion Section for additional information).

The oscillatory CLDN1/HES1 expression has been reported^21, 22^. HES1 transcription repressor is a well-known self-regulated inhibitor and CLDN1 overexpression activates Notch signaling target HES1^14^. These features suggest that oscillatory CLDN1 and HES1 may serve as an activator-inhibitor pair similar to the colon crypts & neuroectoderm patterning Notch-Delta pair^8, 23, 24^. New 2D quantification & mathematical analysis methods just became available to study the effects of chronic stress and RU486 intervention on the differentiation of cell lineage in the crypts with the altered Notch-HES1 axis (Fig. 4)^24^. Glucocorticoid activated NR3C1 is able to inhibit *HES1* promoter transcription and activate *CLDN1* promoter transcription^12^. Oscillatory expression of mRNAs appears to originate from a spinning DNA-chromatin string template. *CLDN1* and *HES1* genes code closely in the opposite direction in the human, rat and mouse genomes as we found in NCBI genome database. The mechanical principles underlying gene transcription provide a theoretical foundation that the observed antagonism between *CLDN1* and *HES1* genes is associated with their proximity and coding in the opposite direction, e.g. they spin in opposite directions while transcribing. Genes can be viewed as pendulums, and mRNA transcribed from spinning gene templates are considered as products of oscillation in a computational model for gene inhibitory auto-regulation^25^. The reciprocal protein levels of CLDN1 and HES1 during differentiation may reflect the behavior of *CLDN1* and *HES1* genes as coupled pendulums proposed in physics. *CLDN1* “knock in” overexpression breaks the dynamic equilibrium of *CLDN1*/*HES1* maintained via the chromatin string, resulting in up-regulation of HES1 in mouse colon and impaired colon epithelium homeostasis^14^. Our observations suggest a novel conception of intra-chromosomal communication between *CLDN1* & *HES1*. It is noteworthy that elucidating the details of *CLDN1/HES1* interactions along the crypt axis will provide insights regarding chromosomal communication and conform to Alan Turing’s concept of the activator/inhibitor gene morphogen pair^23, 26^. Glucocorticoid activated NR3C1 can inhibit *HES1* promoter transcription and activate *CLDN1* promoter transcription (Fig. 2)^12^. Stress-levels of glucocorticoids may impair colon epithelial function by interfering with the *CLDN1-HES1* intra-chromosomal communication via NR3C1/GRE modulation. Our findings are summarized in the model proposed in Fig. 8. The “hardwiring” is similar to what has been described in the latest idealized framework regarding coordinated genomes of patterned cells in tissue, and could be a potential target to test mathematical models with methods being developed for 4D nucleome analysis^27–30^.

## Methods

### Immunohistochemistry (IHC) of human colon mucosal crypts

Adult colonic tissue was collected from deceased donors through the Gift of Life, Michigan (University of Michigan IRB HUM00105750). Specimens were resected from the ascending colon immediately distal to the appendix. After washing is cold DPBS, a 3 cm^2^ full-thickness section was placed in 1.9% buffered formaldehyde. After 12 hours the tissue was formalin-quenched with 3X glycine buffer washes and then stored in 70% Ethyl alcohol for paraffin embedding. Paraffin sections were cut at 5–6 microns and rehydrated to water. Heat-induced epitope retrieval was performed with FLEX TRS High pH Retrieval buffer (pH 9.01) for 20 minutes. After peroxidase blocking, the non-conjugated antibody to HES-1 (#sc-166410; Santa Cruz Biotechnology, Dallas, TX, US) was applied at a dilution of 1:200 to 1:500; and the antibody to NR3C1 (#HPA004248; Sigma-Aldrich, St. Louis, MO, US) was applied at 1:100; both antibodies at room temperature for 60 minutes. A secondary FLEX HRP EnVision System (Dako) was used for detection with a 10 minute DAB chromagen application. Slides were counterstained with Harris Hematoxylin for 5 seconds and then dehydrated and coverslipped. (Kristina Fields, University of Michigan Comprehensive Cancer Center Tissue Core Research Histology and IHC Laboratory). IHC slides were scanned for images on an Aperio AT2 instrument by the University of Michigan Pathology Departmental Slide Scanning Service at 40X, and DAB staining was quantified with Aperio Image Scope (v12.3.2.7001). Full-length mucosal crypts of 225 µm or greater were quantified. We believe that localization of the antibodies to specific regions served as an internal control.

### Caco-2/BBe cell culture

Caco-2/BBe cells are kindly provided by Prof. David E. Smith, Department of Pharmaceutical Sciences, and the University of Michigan. Cells were cultured in DMEM supplemented with 0.01 mg/ml human transferrin, 10% FBS and pen/strep in humidified 37 °C incubator with 5% CO_2_. Cells were seeded at 50–60% density, the medium was replaced with fresh media with vehicle or 500 nM cortisol (GC in human) 1 day after seeding. Then the medium was replaced every 2 or 3 days with vehicle or GC added. HES1 siRNA was transfected to Caco-2/BBe cells and treated with vehicle or 500 nM cortisol for 2 h or 24 h before collected for western blotting analysis. Human HES1 silencing siRNA (#s6922, Life Technologies, Grand Island, NY, US) and RNAiMAX (Life Technologies, Grand Island, NY, US) was used for silencing HES1 in human Caco-2/BBe cells.

### DNA Pull-Down Assay

Rat *CLDN1* promoter GRE/N-box prediction was performed using the online Transcription Element Search System program (http://www.cbil.upenn.edu/cgi-bin/tess/tess). The biotinylated sense and antisense oligonucleotides (random sense sequence: GGGTACAGGGCGTTGATCCGCTGATACTT; GRE (−18 to −47) sense sequence: GGGCGGTTTGGTGTCCCAGAGAACTCCAC; and GRE (−459 to −488) sense sequence: GAATAATCTTGAAGAACAGAGGGGAACAC) were obtained from Invitrogen/Life Technologies (Grand Island, NY, US). Equal amounts of complementary oligonucleotides (10 nmol each in a total volume of 0.2 mL) were heated at 85 °C for 5 min and cooled over 2–3 h to less than 40 °C. Biotin binding streptavidin agarose beads (Sigma-Aldrich, St. Louis, MO, US) were pre-equilibrated by washing once with HEPES buffer (10 mmol/L HEPES, pH 7.5, 1 mmol/L EDTA, 10% glycerol) and re-suspended in one bed-volume of HEPES buffer. Annealed random, GRE (−18 to −47) and GRE (−459 to −488) oligonucleotides (10 nmol in 10 μL) were added to 0.2 mL of 50% slurry plus 500 μL HEPES buffer and incubated at 4 °C for 2 h with constant rotation. DNA bound beads were washed once with HEPES buffer containing 10 mmol/L NaCl and twice more with HEPES buffer without NaCl.

Caco-2/BBe cells were lysed in N-[Tris(hydroxymethyl)methyl]-3-aminopropanesulfonic acid (TAPS) buffer (25 mmol/L TAPS, pH 9.5, 1 mmol/L EDTA, 10% glycerol) with brief vortexing. Cell cytosol was diluted with HEPES buffer to 30% and bound with 1 μmol/L dexamethasone or vehicle (1/40,000 dimethyl sulfoxide) for 2.5 h at 0 °C. After activation for 30 min at 20 °C, cytosols (60 μL) were treated with 20 μL of the 50% slurry containing immobilized DNA oligonucleotides at 4 °C for 16 h with constant rotation. Unbound glucocorticoid receptor was removed by centrifugation, and the samples were washed 3 times with HEPES buffer (1 mL each time) and resuspended in a final volume of 20 μL. Bound proteins were eluted by adding 20 μL of 2× sodium dodecyl sulfate loading buffer (Bio-Rad, Hercules, CA, US) for Western blot analysis.

### Luciferase Assays

Nontumorigenic rat colonic epithelial cell FRC/TEX CL D [D/WT] was kindly provided by Kimberly Rieger-Christ, Ph.D. Sophia Gordon Cancer Center, USA. FRC/TEX cells were cultured in Dulbecco’s modified Eagle medium supplemented with 10% FBS, glutamine, pen/strep, hydrocortisone (0.02 µg/ml), insulin (0.25 µg/ml), transferrin (0.12 µg/ml) and glucose (67.5 µg/ml) in a humidified atmosphere of 10% CO_2_ and 90% air at 37 °C. Rat *CLDN1* promoter plasmids are kindly provided by Daniel G. Cyr, Ph.D. Centre INRS–Institute Armand-Frappier, Canada^31^. All transient transfections of cells were performed using Lipofectamine 3000 (according to the manufacturer’s instructions. 1 μg DNA of the rat *CLDN1* promoter–luciferase construct and 100 ng plasmid pRL-SV40 control vector (used for normalization) were co-transfected into FRC/TEX CL D [D/WT] cells at 50% – 60% confluence. The cells were harvested 24 h after corticosterone (CORT, GC in rodents) treatment, and the activity of firefly or Renilla luciferase was measured using the dual luciferase assay system (Promega, Madison, WI, US). The relative luciferase activity was calculated by normalizing firefly luciferase activity to that of Renilla luciferase. Experiments with each construct were repeated 4 times.

### Animals

Male Sprague–Dawley rats (200–220 g) were obtained from Charles River Laboratories (Wilmington, MA, US). Animals were housed in an animal facility that was maintained at 22 °C with an automatic 12 h light/dark cycle. The animals were given a standard laboratory diet and tap water was available ad libitum. All experimental procedures were performed in accordance with US National Institutes of Health guidelines and were approved by the University Committee on Use and Care of Animals at the University of Michigan.

### Chronic Water Avoidance Stress

Repeated exposure of adult rats to water avoidance (WA) stress was conducted as described previously^3^. The rats were placed on a glass platform in the middle of a tank filled with water (25 °C) to 1 cm below the height of the platform. The animals were maintained in the tank for 1 h in the morning daily for 10 consecutive days. The control (sham-stress) rats were placed similarly for 1 h daily for 10 days in a tank without water.

### qPCR

Total RNA was isolated epithelial cells scrapped from colon lumen using the Trizol (Life Technologies, Grand Island, NY, US) and RNeasy kit (Qiagen, Hilden, Germany). qPCR was performed using the Bio-Rad iScript One-Step Reverse-Transcription PCR Kit with SYBR Green (Bio-Rad, Hercules, CA, US) using the following specific primers from Integrated DNA Technologies (Coralville, IA, US): NR3C1: forward- GCGTCAAGTGATTGCAGCAGTGAA, reverse-GCAAAGCAGAGCAGGTTTCCACTT; CLDN1: forward-AGGCAACCAGAGCCTTGATGGTAA, reverse-CATGCACTTCATGCCAATGGTGGA; GAPDH: forward-ACAAGATGGTGAAGGTCGGTGTGA, reverse-AGCTTCCCATTCTCAGCCTTGACT.

### Isolation of colon crypts

Rats were sacrificed and colon (1.5–5 cm from anus) were taken and lumen side were inversed to the outside and incubated in DPBS (without Ca^2+^ & Mg^2+^) containing 4 mM EDTA and 0.5 mM DTT in 15 ml Tubes, after 10–15 rotation at room temperature, crypts detached were collected by 50 g spin for 2 min at 4 °C. Crypts were snap frozen in liquid nitrogen after brief wash with ice-cold PBS and were kept at −80 °C before use. Human colon crypts were isolated with the same method with reduced volume.

### Western Blotting

For immunoblot analysis, isolated colon crypts or Caco-2/BBe cells scraped off from the dish were lysed with NP40 lysis buffer (50 mmol/L TrisHCl, 150 mmol/L NaCl, 1% NP40, pH 8.0) supplied with protease inhibitor cocktail (Roche, Indianapolis, IN, USA) on ice, we used 1 ml syringe to pass the lysates through 27 Gauge needle 6 times and samples were kept on ice for 20 min lysis before 5 min 6000 rpm centrifugation at 4 °C. Supernatants were collected for SDS-PAGE analysis. The following primary antibodies were used: anti- CLDN1 antibody (1:10000; 51–9000; Thermo Fisher, Waltham, MA, US), anti HES1 antibody (1:10000; sc-25392, Santa Cruz, Dallas, TX, US), anti- Notch1 antibody (1:2000; #3608; Cell Signaling Technology, Danvers, MA, US), anti- Cleaved Notch1 antibody (1:2000; #4147; Cell Signaling Technology, Danvers, MA, US), anti- NR3C1 antibody (1:10000; #3660; Cell Signaling Technology, Danvers, MA, US), anti-β-actin antibody (1:20000; A1978, Sigma-Aldrich, St. Louis, MO, US) overnight at 4 °C. The corresponding immunoblot bands were scanned at 1200 dots per inch and analyzed with ImageJ.

### Chromatin Immunoprecipitation (ChIP) Assay

ChIP was performed using a chromatin immunoprecipitation kit (Epigentek, Farmingdale, NY, US). In brief, rat colon crypts and Caco-2/BBe cells were cross-linked using 1% formaldehyde and terminated by incubation with 0.125 mol/L glycine for 5 min. The cell lysate was incubated for 10 min at 4 °C and the crude nuclear extract was collected by centrifugation at 600 g for 5 min at 4 °C. The DNA was sonicated to random fragments between 200 and 500 bps. The chromatin was subjected to immunoprecipitation using the following antibodies: NR3C1 (#3660; Cell Signaling Technology, Danvers, MA, US), HES1 (#sc-1004; Santa Cruz Biotechnology, Dallas, TX, US). Normal rabbit IgG was used as a control. DNA was eluted in elution buffer and used for PCR amplification. Primers for rat *CLDN1* promoter: forward-GGACCCTTGTGGGGATTTG, reverse- CCAGGAGGTTAGCGCTGATAC and human *CLDN1* promoter: forward-GATAATTGGAGTGAATGAATGAAAAG, reverse-GTTTCAGGGCGGCTCAC are bought from Life Technologies (Grand Island, NY, US).

### Measurement of the Transepithelial Electrical Resistance (TEER)

Caco-2/BBe cells were cultured with low density (50–60% before Trypsin digestion) or 500 nM cortisol added to the media with regular density (90–100% before Trypsin digestion) for 10 passages. Measurements were performed during 21-day differentiation with control, low density or cortisol trained cells seeded with 50% density in transwells in 24 well plates and the standard medium was replaced every other day after transepithelial electrical resistance (TEER) measurements. TEER measurements were performed using an EVOM2, Epithelial Voltohmmeter for TEER (World Precision Instruments., Sarasota, FL, US) following manufacturer’s video instructions in product page as a measurement for Caco-2/BBe monolayer integrity.

### Quantification of NR3C1 and HES1 in Caco-2/BBe cells before and after differentiation

Caco-2/BBe cells were cultured on glass coverclips for 1 day or 21 days and fixed with 4% PFA and immuno-stained with Alexa 647 direct-labeled HES1 antibody (1:200; ab196577, Abcam, Cambridge, UK), NR3C1 antibody (1:400; #3660, Cell Signaling Technology, Danvers, MA, US) and Alexa 594 secondary antibody (1:600; Thermo Fisher, Waltham, MA, USA). Nuclei were labeled with DAPI (4,6-diamidino-2-phenylindole). 1024 × 1024, 16-bit 3D confocal images were acquired using a Zeiss LSM 710 laser scanning confocal microscope with a 63x Plan-Apochromat 1.4na DIC objective. For HES1 quantification, images were taken keeping the same laser settings for HES1 channel for all samples. Three 1024 × 1024 Day21 3D images and seven 1024 × 1024 Day1 3D images were collected. Maximum intensity projections of 3D image stacks were analyzed to compare average intensities of HES1 in Day1 and Day 21 Caco-2/BBe cells using Fiji^32^. 8-bit images were created and the DAPI channel was used to create nuclei masks. Overlapping nuclei and nuclei on the edge of the image were then manually eliminated (Figure S5). These masks were redirected to the corresponding HES1 and NR3C1 channels to analyze mean intensities of HES1 and NR3C1 within the nucleus. T- test was performed on the mean intensities per nuclei of 100 total nuclei for each sample set (Day1 & Day21).

## Electronic supplementary material

Supplementary Information
