# Supplementary material for: Chronic stress and intestinal barrier dysfunction: Glucocorticoid receptor and transcription repressor HES1 regulate tight junction protein Claudin-1 promoter
Source: Sci Rep. 2017 Jul 3;7:4502. doi: 10.1038/s41598-017-04755-w (PMC5495803; doi:10.1038/s41598-017-04755-w)

Chronic stress and intestinal barrier dysfunction: Glucocorticoid receptor and transcription repressor HES1 regulate tight junction protein Claudin-1 promoter

Gen Zheng[1,*], Gordon Victor Fon[2], Walter Meixner[2], Amy Creekmore[1], Ye Zong[3], Michael Dame[4], Justin Colacino[5], Priya H. Dedhia[6], Shuangsong Hong[1] and John W. Wiley[1]

[1]University of Michigan Medical School, Division of Gastroenterology, Department of Internal Medicine, Ann Arbor, 48109, USA

[2]University of Michigan Medical School, Department of Computational Medicine and Bioinformatics, Ann Arbor, 48109, USA

[3]Beijing Friendship Hospital Affiliated to Capital Medical University, Department of Gastroenterology and Hepatology, Beijing, 100050, China

[4]University of Michigan Medical School, Department of Pathology, Ann Arbor, 48109, USA

[5]University of Michigan School of Public Health, Department of Environmental Health Sciences, Ann Arbor, 48109, USA

[6]Department of Surgery, University of Michigan, Ann Arbor, MI, 48109, USA

[*]zhenggen@umich.edu

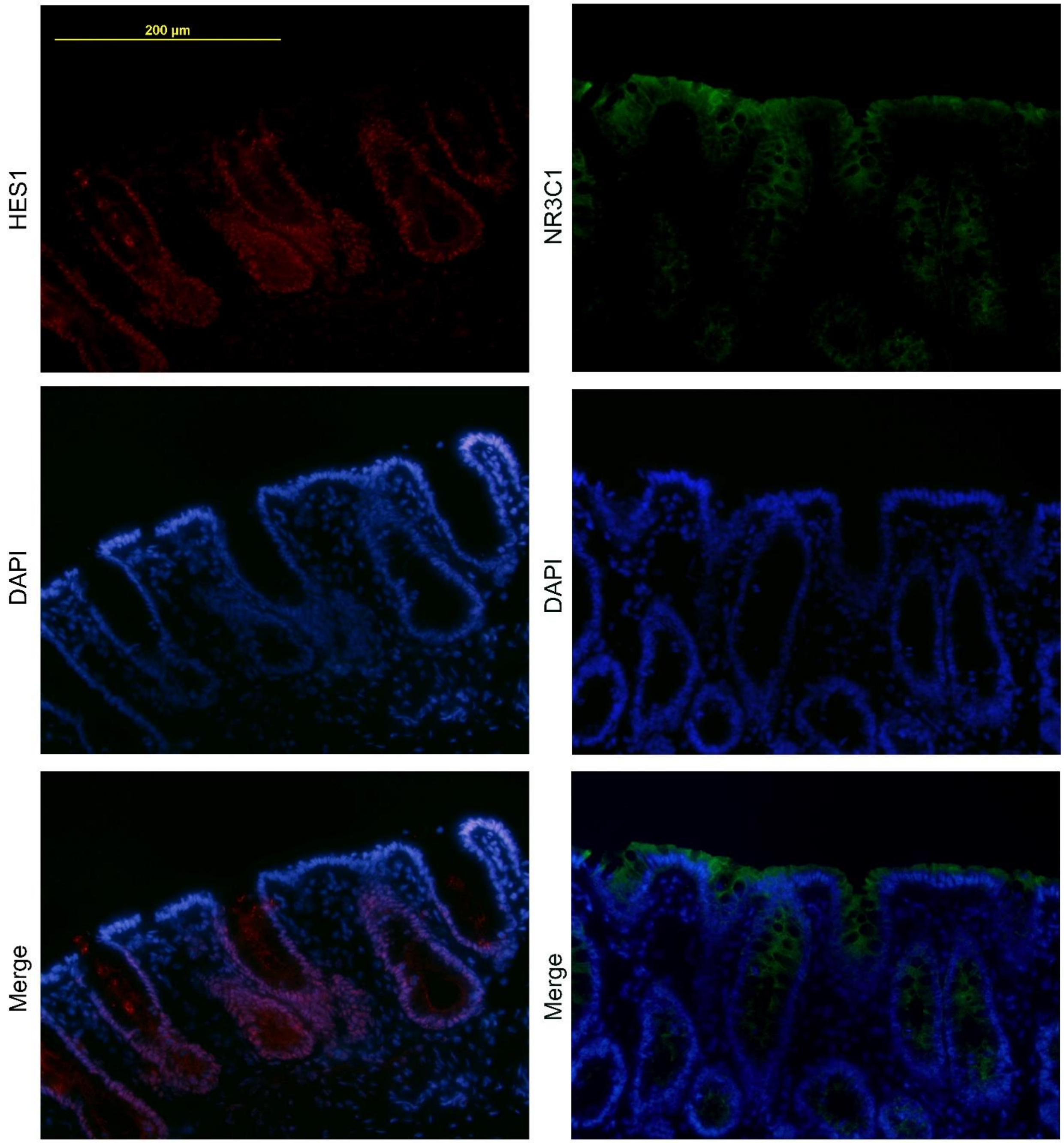

Figure S1. HES1 and NR3C1 immunostaining in rat colon crypts.

Rat colon sections (10 µm thickness) from healthy control rats were stained with HES1 antibody and Alexa 594 secondary antibody / NR3C1 antibody and Alexa 488 secondary antibody. Representative images are shown. The majority of HES1 positive cells are located in the bottoms of the crypts, whereas NR3C1 immunofluorescent signals are higher in the apex of the crypts.

```
                                                            HES1
                                                           ------
                  TCAAAAGTCATCCGCGACCACGCAGGAGGCCTGCGAGGACCCTTGTGGGGATTTGGCCAC

                        HES1                                    SP1       TATA box
                       ------                           --  --------   -------
Rat    -95        GAAACCACCAGGGGGCGCTGCA----TGCCGGG----AGG--GGCGGAGCTGCTTTAAAT  -46
                  |||||| |||||||||||| |     |||||||    |||  ||||||||||||||||||
human  -281       GAAACCGCCAGGGGGCGCTCCCCGGCTGCCGGGGCTGAGGCGGGCGGAGCTGCTTTAAAT  -222

                              SP1      NR3C1
                         ------------ --------
Rat    -45        CGCAGCGCCGGGCGGTTTGGTGTCCCAG----AGAAC---------TCCACCACTTCTCA  +2
                  ||| |||||  ||||||  | ||| |||     || |          || |  |||||
Human  -221       CGCGGCGCCCAGCGGTTCTGCGTCTCAGTTCCCGAGCCTGGGAGCAACCGCAGCTTCTAG  -162
```

Figure S2. Alignment of rat/human *CLDN1* promoters.

a

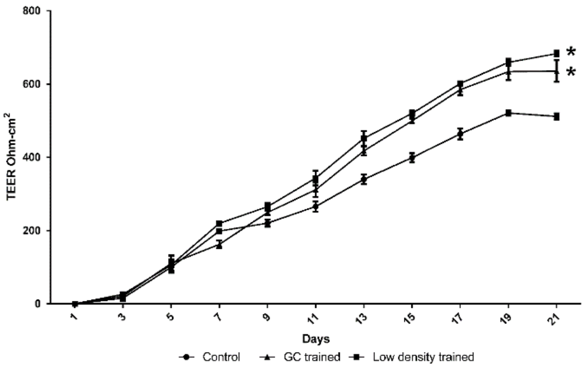

b

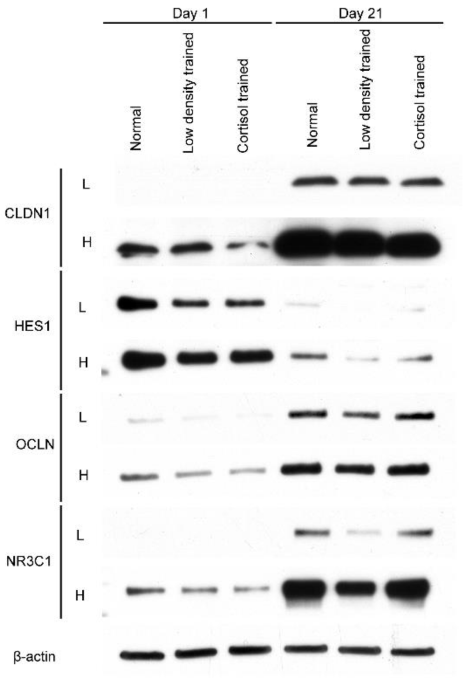

Figure S3. Transmembrane electrical resistance (TEER) and expression of tight junctions in GC/low-density culture trained Caco-2/BBe cells during 21-day differentiation.

a. Caco-2/BBe cells were cultured with low-density or medium supplemented with GC (500 nM cortisol) with regular density for 10 passages. After "training", cells were seeded in transwells in 24 well plate. The medium was replaced with same medium every 2 days and transmembrane electrical resistance (TEER) was measured before medium replacement. GC training showed an increase of TEER similar to low-density training (n=4; two-tailed test; *, $P<0.05$).

b. Western blotting analysis of control and GC/low-density culture trained Caco-2/BBe cells before and after differentiation. High (H) exposure and low (L) exposure films are shown together to cover the broad dynamic range.

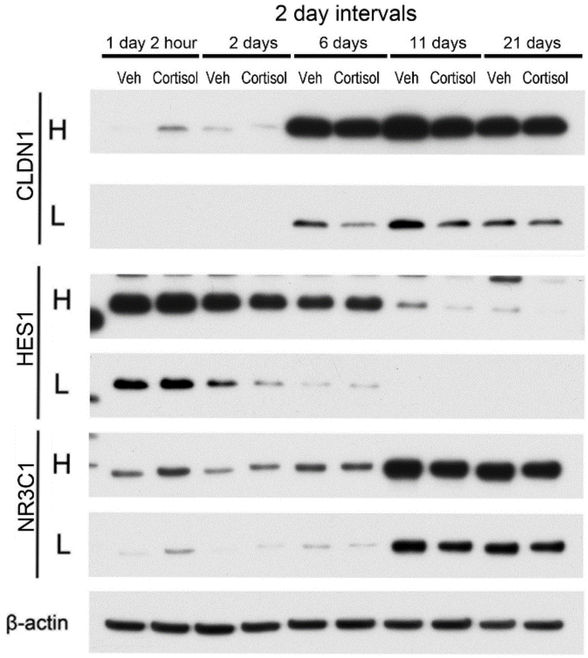

Figure S4. 2-day GC + medium replacement intervals demonstrated similar but shifted wave-like dynamics along time axis compared to that observed with 3-day intervals.

1 day after Caco-2/BBe cells are seeded in 6 well plates, the medium was replaced with fresh medium supplemented with vehicle or GC (500 nM cortisol), cells were harvested after 2 h treatment or 2, 6, 11, 21 days after seeding, the medium was replaced every 2 days. Cells were harvested for Western blotting analysis for protein expression and. High (H) exposure and low (L) exposure films are shown together to cover the broad dynamic range.

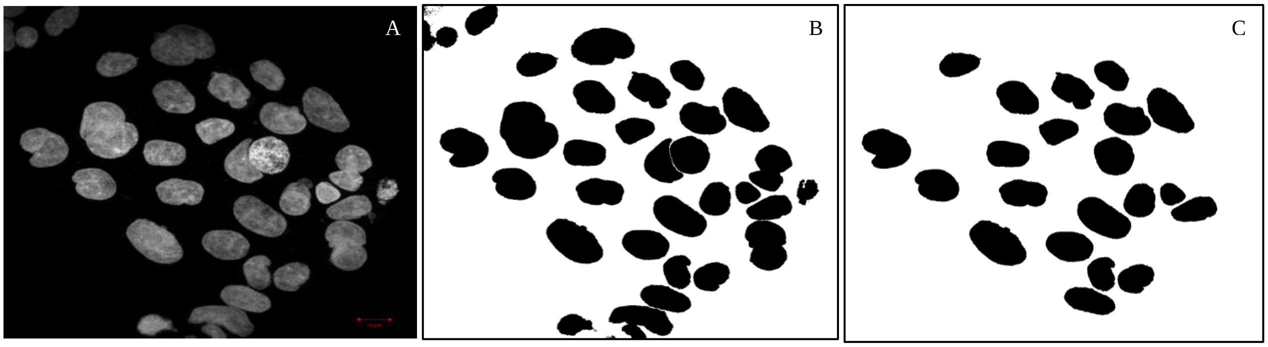

Figure S5. Workflow of cell segmentation in single cell resolution for HES1/NR3C1 quantification

(A) Original DAPI image (B) nuclei mask (C) refined masks, overlapping and boundary nuclei eliminated.

Supplementary Info for Figure 4

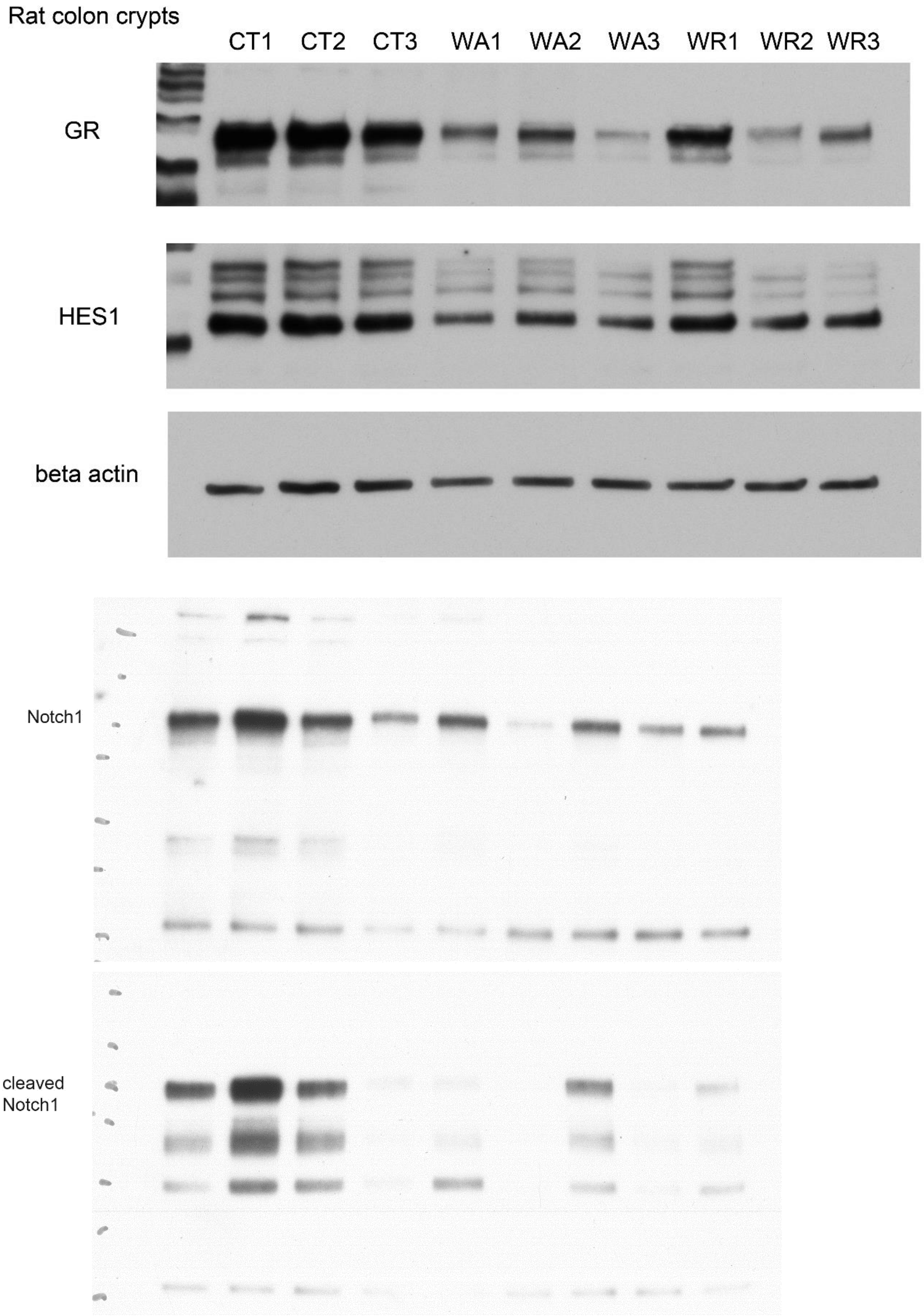

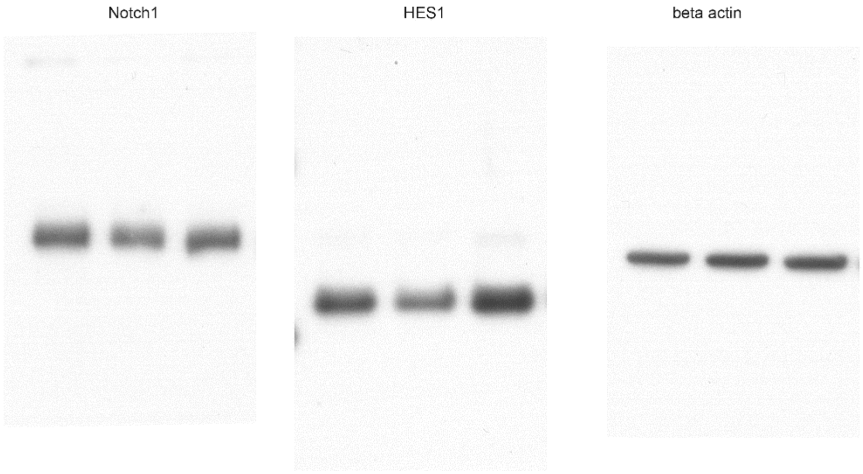

Supplementary Info for Figure 5

Caco-2 BBE cell differentiation media replaced every 3 days with cortiosol

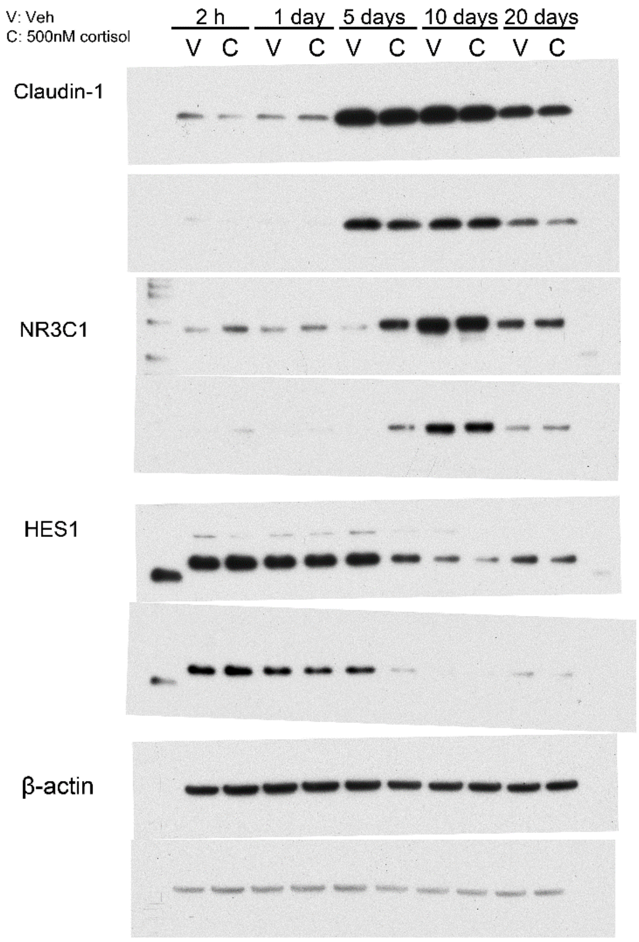

Supplementary Info for Figure 6

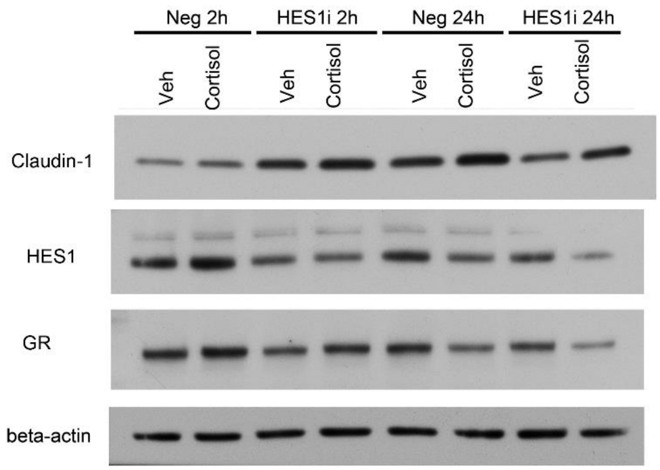

Supplement: Supplementary file 1 — Supplementary Information [file 41598_2017_4755_MOESM1_ESM.pdf]
